# Supplementary material for: Habitat ephemerality affects the evolution of contrasting growth strategies and cannibalism in anuran larvae
Source: PeerJ. 2021 Sep 13;9:e12172. doi: 10.7717/peerj.12172 (PMC8445080; doi:10.7717/peerj.12172)
Supplement: Supplemental Information 1 [file peerj-09-12172-s001.pdf]

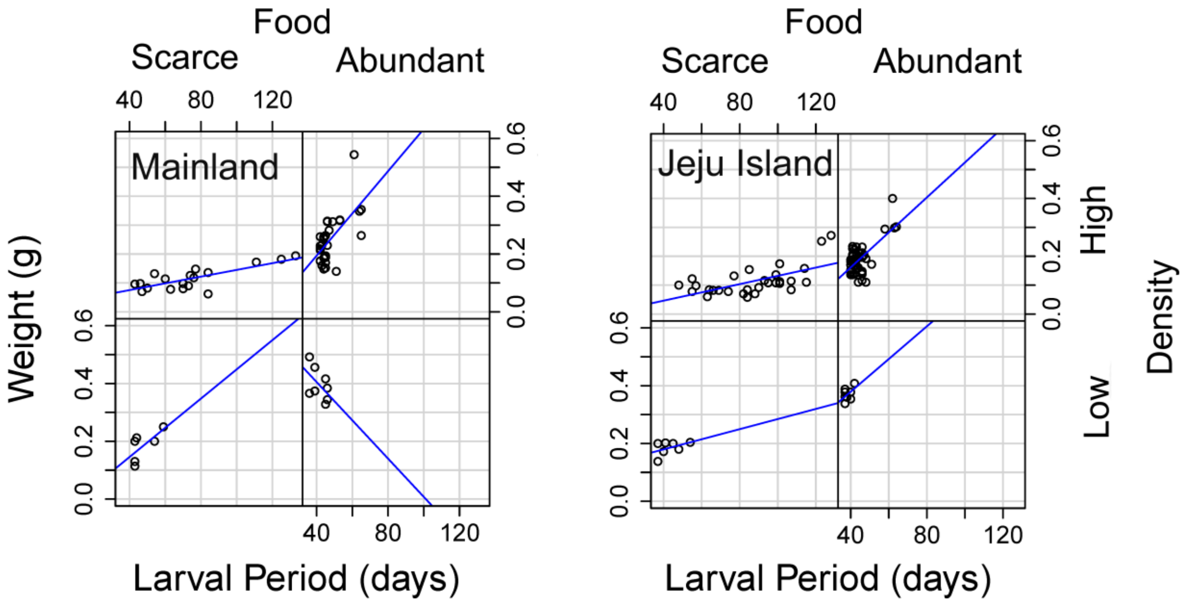

Figure S1. The correlation between larval period and weight at metamorphosis within each treatment group. There were positive correlations between larval period and weight at metamorphosis except in mainland frogs when they were exposed to abundant food and low density. Lines show linearly predicted values within each group.
